# Papillary Adenocarcinoma of the Common Bile Duct

**DOI:** 10.1155/1995/86293

**Published:** 1995

**Authors:** O. Arioğul, B. H. Güvenç, A. Emre, K. Acarli, A. Alper, A. Ökten

**Affiliations:** 1 Department of Hepatobiliary Surgery and Hepatology of Istanbul Medical Faculty Çapa Istanbul Turkey; 2 Zincirlikuyu cad.Dağ apt. 72/6 80600 Etiler Istanbul Turkey

## Abstract

Five patients with papillary adenocarcinoma of the common bile duct (CBD) are described. These are rare tumors and make up 5% of all malignant tumors of the biliary tract. The symptoms and signs at the time of initial diagnosis resemble benign obstructive lesions of the bile ducts. The tumor is soft, less invasive to adjacent tissues and tends to grow into the lumen. The early onset of the symptoms results in early intervention, with a better prognosis. Two of our patients are doing well after two and four years, where as three others were readmitted with recurrent disease.


*HPB Surgery*, 1995, Vol. 8, pp. 245–248
Reprints available directly from the publisher
Photocopying permitted by license only

© 1995 OPA (Overseas Publishers Association)
Amsterdam B.V. Published in The Netherlands
by Harwood Academic Publishers GmbH
Printed in Malaysia

### Papillary Adenocarcinoma of the Common Bile Duct

O. ARIOĞUL*, B. H. GÜVENÇ, A. EMRE, K. ACARLI, A. ALPER and A. ÖKTEN

Department of Hepatobiliary Surgery and Hepatology of Istanbul Medical Faculty,
Çapa, Istanbul, Turkey

*(Received February 10, 1994)*

Five patients with papillary adenocarcinoma of the common bile duct (CBD) are described. These are rare tumors and make up 5% of all malignant tumors of the biliary tract. The symptoms and signs at the time of initial diagnosis resemble benign obstructive lesions of the bile ducts. The tumor is soft, less invasive to adjacent tissues and tends to grow into the lumen. The early onset of the symptoms results in early intervention, with a better prognosis. Two of our patients are doing well after two and four years, where as three others were readmitted with recurrent disease.

KEY WORDS: Common bile duct papillary adenocarcinoma

### INTRODUCTION

Papillary adenocarcinoma of the CBD is regarded as an uncommon tumor with a more favourable prognosis and better survival compared with other carcinomas of the extrahepatic bile ducts[1–4]. These tumors are raised, soft, frond-like lesions. They are covered with epithelium and grow into the lumen of the bile duct producing incomplete obstruction, with signs and symptoms resembling bile duct stones or strictures[2–4].

It seems that, detection of early intraluminal growth is followed by early surgical resection with a relatively better survival rate. In this report five cases are presented and current methods of diagnosis and treatment of papillary adenocarcinoma of the CBD are reviewed.

### CASE REPORTS

Case 1: A 45 year old male was referred with a six-month history of jaundice, pruritus, weight loss, chills and fever, accompanied by right upper quadrant pain. He had been operated on twice elsewhere without any apparent diagnosis. Percutaneous transhepatic cholangiography (PTC) showed a tumor mass in the proximal CBD.

Peroperative findings were a papillamatous, fround-like, soft tumoral mass pouring out of the transected CBD, and a cholestatic liver. The tumor was identified as a papillary adenocarcinoma on frozen section. A hepatico-duodenostomy was performed following resection of the CBD. The patient was discharged on the 21st postoperative day. He was readmitted 2.5 years later with obstructive jaundice, was re-explored and found to have a tumor infiltrating the hilum of the liver. A bilio-digestive anastomosis was established using the segment III duct. The patient died three months after the operation (Table 1).

Case 2: A sixty-one year-old male complained of weight loss, jaundice, pruritus, high fever, fatigue and right upper quadrant pain lasting nine months. Physical examination showed jaundice with mild hepatomegaly. Ultrasonography (US) demonstrated dilated intrahepatic ducts with a tumor mass in the proximal part of the CBD. A papillary tumor was found at the level of the cystic duct junction peroperatively. Tumor was identified as papillary adenocarcinoma of the CBD. A Roux en-Y hepatico-jejunostomy was performed following CBD resection. The patient was discharged on the 26th post-operative day, only to be readmitted two months later, he was

* Address for correspondence to: Dr. O. Arıoğul, Zincirlikuyu cad.Dağ apt. 72/6, 80600 Etiler, Istanbul, Turkey.

**Table 1** Patient characteristics, intervention and outcome

| AGE | SEX | DUR mo. | PAI | JAU | PRU | W.L. | FEV | PREV. OP. | TB/DB (mg/dL) | A.P. (B.L.U.) | PTC | US | OPERATION | PROGNOSIS |
|---|---|---|---|---|---|---|---|---|---|---|---|---|---|---|
| 45 | M | 6 | + | + | + | + | + | TWICE | 8.9/6.5 | 14 | Tumor mass at hilar level | – | Hepatico duodenostomy | Expired 3 years postop |
| 61 | M | 9 | + | + | – | + | – | – | 4.7/3.6 | 6.4 | – | Tumor mass in distal CBD | R-en Y Hep.-jejunostomy | Peritoneal met. in 2 mo. |
| 64 | M | 6 | + | + | + | + | – | – | 11/9.2 | 8.7 | Filling defect in the CBD | – | Hepatico-duodenostomy | Doing well after 4 years |
| 40 | F | 4 | – | + | + | – | – | – | 4.5/3.8 | 6.6 | Left intrahep. duct not seen | – | R-en Y Hep.-jejunostomy | Liver met. after 3 years |
| 65 | F | 5 | + | + | + | – | + | Once | 4.6/4.6 | 6.3 | Filling defect in the CBD | – | Hepatico-duodenostomy | Doing well after 2 years |

DUR: DURATION; PAI: PAIN; JAU: JAUNDICE; PRU: PRURITUS, W.L.: WEIGHT LOSS; FEV: FEVER; PREV. OP.: PREVIOUS OPERATION; TB/DB: TOTAL BILIRUBIN/DIRECT BILIRUBIN; A.P.: ALKALINE PHOSPHATASE

reexplored and found to have carcinomatosis peritonei.

Case 3: A sixty-four year old male had complained of jaundice, pruritus, fever, fatigue, weight loss and postprandial abdominal pain for six months. PTC showed a filling defect in the CBD. At operation, the CBD was found to be 30 mm in diameter with a soft amorphous polypoid tumoral mass filling the lumen, just below the cystic duct junction. Frozen section showed papillary adenocarcinoma. A Hepatico-duodenostomy was performed following CBD resection and cholecystectomy; an enlarged suprapancreatic lymph node was also removed. Histo-pathologic examination showed papillary adenocarcinoma with lymph node metastasis. The patient has been well for four years postoperatively.

Case 4: A forty-year old female complained of jaundice, pruritus and fever for four months. PTC showed dilated right intrahepatic ducts, the left intrahepatic ducts were not seen, there was partial obstruction of the CBD beginning at the cystic duct junction and extending upward to the left hepatic duct. At operation the CBD was found to be 25 mm in diameter, containing a papillary tumor, which was identified as a papillary adeno-carcinoma by frozen section. Hepatic ducts, along with the CBD were removed. Reconstruction was done by a Roux en-Y hepatico-jejunostomy. The patient was discharged on the 23rd postoperative day and now has liver metastasis three years after the operation.

Case 5: A sixty-five year old female presented with jaundice, fever, abdominal pain and pruritus for five months. She had undergone a cholecystectomy five years ago. On physical examination there was marked jaundice and a tender right upper quadrant mass. A filling defect was detected in the CBD by PTC. Operative findings were, a soft palpable mass within the CBD and marked cholestasis in the liver. Frozen section showed papillary adenocarcinoma. A hepatico-duodenostomy was performed following resection of the CBD. The patient has been well for two years postoperatively.

### DISCUSSION

Tumors of the CBD are rare neoplasms. Papillary adenocarcinomas constitute 5% of all malignant tumors of the biliary tract. These tumors are easily resected, with a better prognosis when compared with the non-papillary types[1,3–6]

Biliary tract tumors are seen mostly during the sixth decade[7] and in older patients papillary tumors of the bile ducts should be considered, as a cause of jaundice. Papillary adenocarcinomas often lead to early signs and symptoms of obstructive jaundice resembling benign biliary tract disorders. The tumor is soft and fragile in nature, it allows bile to pass intermittently; consequently serum bilirubin levels are lower in comparison to the non-papillary types[1–3]. All of our patients were admitted with cholangitis. The mean bilirubin level in our five cases was 114 μmol/L, while it is calculated to be 231 μmol/L in nonpapillary tumors. Even with present day imaging techniques, there are still cases that undergo repeated operations, until the tumor is found. Our first case is such an example[1,3,7].

The most commonly used diagnostic techniques in biliary tract disease are US and PTC. Papillary tumor can be seen as a silhouette growing into the lumen, adjacent to the wall[8]. Recently, ERCP and bile duct drainage fluid cytology has been introduced into clinical use, and has proved to be quite effective[9].

Early diagnosis has considerable importance in the treatment of papillary tumors of the CBD. The wall of the bile duct is often normal in the early stage and in such cases the tumor can simply be overlooked during exploration.

As for treatment, conservative resection of the CBD with either primary reanastomosis or enteric drainage is recommended[1,7,8,10]. We were able to resect the soft tumor mass along with the common bile duct in all of our cases. We have not seen any invasion of the tumor beyond the lumen of the CBD. Intraluminal growth of the tumor and lack of mural invasion has enabled us to perform these resections. The overall resectability rate of all bile duct tumors does not exceed 28 to 32% in recent studies[6,11,12]. In those cases where the tumor is located in the distal CBD, the need for a duodenopancreatectomy along with the common bile duct will be unavoidable, a situation that we have not yet confronted. One of our patients died six months after surgical intervention with carcinomatosis peritonei. One of our patients survived three years, another two were alive two to four years postoperatively.

Papillary type tumors show a better survival rate when compared to nonpapillary types, in all series[2–5,7]. Okuda[8] reported successful resection in all of the four cases of papillary tumors, where as resectability rate fell to 10% in the other tumor types.

Histologically papillary carcinomas of the bile duct are composed of characteristic cells which vary from round to polyhedral configuration and are arranged in a papillary like pattern (Fig. 1)[3,9,13]. The differential diagnosis between moderate to poorly differentiated papillary duct carcinomas and other bile duct carcinomas however, is said to be impossible[9]. Whether those who survive for long periods without recurrence, may

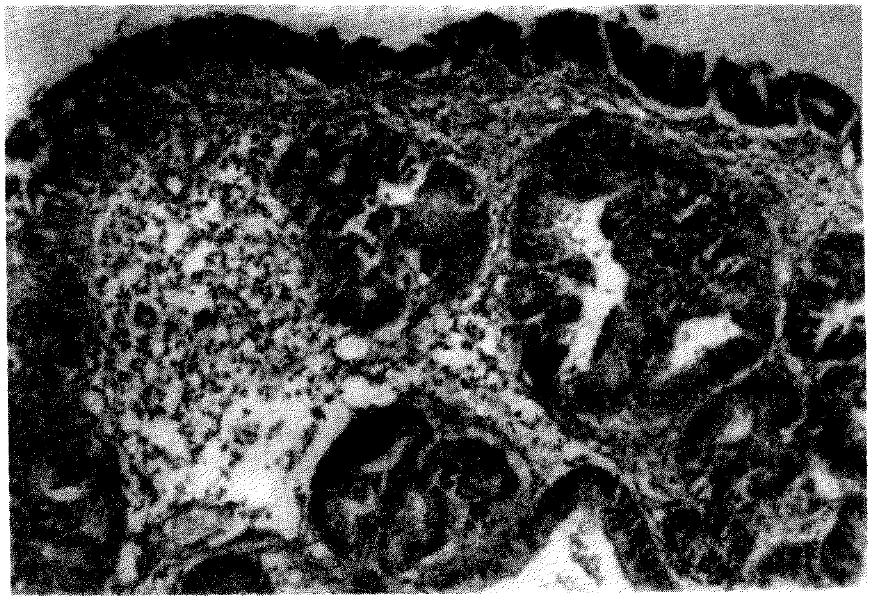

**Figure 1** The cells are arranged in a papillary pattern showing tumoral infiltration with a typical nuclei under the epithelial surface of the tissue (Magnification X 125).

actually be cases of benign papillomas with minor foci of early adenocarcinoma is a matter of discussion. There are reports of papillary type tumors showing malignant degeneration[12].

In conclusion, papillary tumors of the common bile duct should be born in mind in the differential diagnosis of benign lesions of the biliary tract. The resectability of these tumors when recognised at an early stage, points to the importance of this differential diagnosis.

### REFERENCES

1. Cattell, R. B., Braasch, J. W. and Kahn, F. (1962) Polypoid epithelial tumors of the bile duct. *New Eng. J. Med.*, **266**, 57–61.
2. Longmire, W. P. Jr., McArthur, M. S., Bastonnis, E. A., Hiatt, J. (1973) Carcinoma of the extrahepatic biliary tract. *Ann. Surg.*, **178**, 333–337.
3. Malvy, P., Le Neel, J. C., Nomballis, M. F., Le Borgne, J. and Visset, J. (1976) Une forme rare de cancer de la voie biliaire principle:l'epithelioma papillaire. *J. Chir.*, **112**, 15–24.
4. Sako, K., Seitzinger, G. L. and Garside, E. (1957) Review of literature and report of six cases. Carcinoma of the extrahepatic bile ducts. *Surgery*, **41**, 416–419.
5. Styne, P., Warren, G. H., Kumpe, D. A., Halgrimson, C. and Kern Jr. F. (1986) Obstructive cholangitis secondary to mucus secreted by a solitary papillary bile tumor. *Gastroenterology*, **90**, 748–753.
6. Orloff, M. J. and Marrassi, N. P. (1985) Tumors of the extrahepatic bile ducts. Bochus Gastroenterology vol. 6 Ed. in chief: J. Berk., W. B. Saunders Company, Philadelphia, pp. 3771–3778.
7. Chitwood, W. R., Meyers, W. C., Heaston, D. K., Herskovic, A. M., McLeod, M. E. and Jones, R. S. (1982) Diagnosis and treatment of primary extrahepatic bile duct tumors. *Am. J. Surg.*, **143**, 99–106.
8. Okuda, K., Onto, M. and Tsuchiya, Y. (1988) The role of Ultrasound, percutaneous transhepatic cholangiography, computed tomographic scanning and magnetic resonance imaging in the preoperative assessment of bile duct cancer. *World J. Surg.*, **12**, 18–26.
9. Ahsan, N. and Bergman, J. J. (1988) Papillary carcinoma of the common bile duct:Diagnosis by bile drainage cytology. *Acta Cytologica*, **32**, 471–474.
10. Neumann, R. D., Livolsi, V. A., Rosenthal, N. S., Burreel, M. and Ball, T. (1976) Adenocarcinoma in biliary papillomatosis *Gastroenterology.*, **70**, 779–782.
11. Nakayama, F., Miyazaki, K. and Nagafuchi, K. (1988) Radical surgery for middle and distal thirds bile duct cancer. *World J. Surg.*, **12**, 60–63.
12. Yamaguchi, K., Enjoji, M. and Nakayama, F. (1988) Cancer of extrahepatic bile ducts; Clinicopathologic study of Immunochemistry for CEA, CA 19–9 and P21. *World J. Surg.*, **12**, 11–17.
13. Todoroki, T., Okamura, T., Fukao, K. Nishimura, A., Otsu, H., Sato, H. and Iwasaki, Y. (1980) Gross appearance of carcinoma of the main hepatic duct and it prognosis. *Surg. Gynecol. Obstet.*, **150**, 33–40.

